# Cysteine Biosynthesis in *Campylobacter jejuni*: Substrate Specificity of CysM and the Dualism of Sulfide

**DOI:** 10.3390/biom13010086

**Published:** 2022-12-31

**Authors:** Noah Hitchcock, David J. Kelly, Andrew Hitchcock, Aidan J. Taylor

**Affiliations:** 1 School of Biosciences, University of Sheffield, Sheffield S10 2TN, UK; 2 School of Biological Sciences, University of Reading, Reading RG6 6AX, UK

**Keywords:** *Campylobacter jejuni*, cysteine synthase, CysM, sulfide, thiosulfate, sulfur, oxidase

## Abstract

*Campylobacter jejuni* is a highly successful enteric pathogen with a small, host-adapted genome (1.64 Mbp, ~1650 coding genes). As a result, *C. jejuni* has limited capacity in numerous metabolic pathways, including sulfur metabolism. Unable to utilise ionic sulfur, *C. jejuni* relies on the uptake of exogenous cysteine and its derivatives for its supply of this essential amino acid. Cysteine can also be synthesized de novo by the sole cysteine synthase, CysM. In this study, we explored the substrate specificity of purified *C. jejuni* CysM and define it as an O-acetyl-L-serine sulfhydrylase with an almost absolute preference for sulfide as sulfur donor. Sulfide is produced in abundance in the intestinal niche *C. jejuni* colonises, yet sulfide is generally viewed as highly toxic to bacteria. We conducted a series of growth experiments in sulfur-limited media and demonstrate that sulfide is an excellent sulfur source for *C. jejuni* at physiologically relevant concentrations, combating the view of sulfide as a purely deleterious compound to bacteria. Nonetheless, *C. jejuni* is indeed inhibited by elevated concentrations of sulfide and we sought to understand the targets involved. Surprisingly, we found that inactivation of the sulfide-sensitive primary terminal oxidase, the *cbb*_3_-type cytochrome *c* oxidase CcoNOPQ, did not explain the majority of growth inhibition by sulfide. Therefore, further work is required to reveal the cellular targets responsible for sulfide toxicity in *C. jejuni*.

## 1. Introduction

*Campylobacter jejuni* is a microaerophilic Gram-negative enteric pathogen, most commonly associated with avian species, including commercial broiler chickens, which are the most common source of human infection. Pathogenic *Campylobacter* species are accountable for 400–500 million human infection cases worldwide each year [1]. Specifically, *C. jejuni* is a zoonotic pathogen that causes acute gastrointestinal infections, termed campylobacteriosis. Infections primarily arise through the consumption of contaminated poultry meat [2]. The symptoms of campylobacteriosis include vomiting, diarrhoea, abdominal pain and fever, which are typically self-limiting with a recovery time of 1–2 weeks [3]. 

Sulfur is an essential element for life, most notably required for the biosynthesis of the amino acid cysteine. Cysteine can be interconverted with the other sulfur containing amino acid, methionine, and is itself a crucial residue for the formation of iron-sulfur clusters found in various proteins [4]. Additionally, sulfur is the active moiety in reducing agents such as glutathione (GSH, Glu-Cys-Gly) and in essential cofactors such as lipoic acid, coenzyme A and other low molecular weight thiols, some of which are cytoplasmic antioxidants that defend against oxidative damage [5]. 

During cysteine limitation, *Escherichia coli* activates the sulfate assimilation pathway through CysB, a positive regulator of gene expression for the cysteine synthesis regulon [6]. The sulfate assimilation pathway reduces sulfate taken up by the cell to ultimately form hydrogen sulfide, required for cysteine synthesis. However, *C. jejuni* lacks several of the enzymes in the sulfate assimilation pathway, in addition to any homologues of CysB or the typical bacterial ABC-type sulfate transporter [4]. The sulfur metabolism pathways of *C. jejuni* are therefore markedly limited, and the cell requires alternative exogenous sources of sulfur for growth. *C. jejuni* does not grow on sulfate or sulfite but can utilise thiosulfate, sulfide, glutathione and free reduced cysteine as sulfur sources [7,8]. Cysteine containing di- and tri-peptides are also a potential sulfur source, including Gly-Cys, Glu-Cys and the cysteine-disulfide cystine (Cys-Cys) [4,8]. In the absence of exogenous cysteine containing compounds, cysteine must be generated de novo by cysteine synthase. 

**Figure 1:**
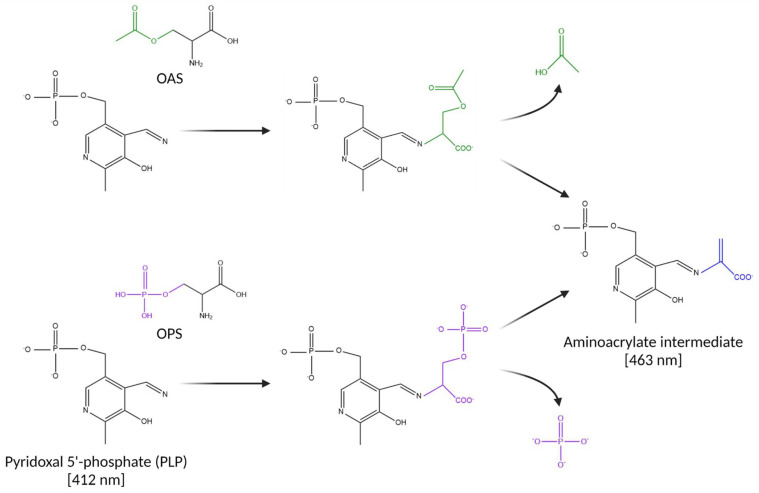
First half-reaction of PLP-dependent cysteine synthase. The enzyme bound PLP cofactor forms an internal Schiff base with a catalytic lysine residue which transfers to the sulfur acceptor (either OAS or OPS) to form an intermediate which subsequently releases acetate or phosphate (for OAS and OPS, respectively) through a β-elimination event. Subsequent proton extraction from the α position by the catalytic lysine resolves the covalently bound α-aminoacrylate intermediate form. Resting PLP and the aminoacrylate intermediate can be differentiated spectrophotometrically by their respective absorbance maximum of 412 and 463 nm.

**Figure 2:**
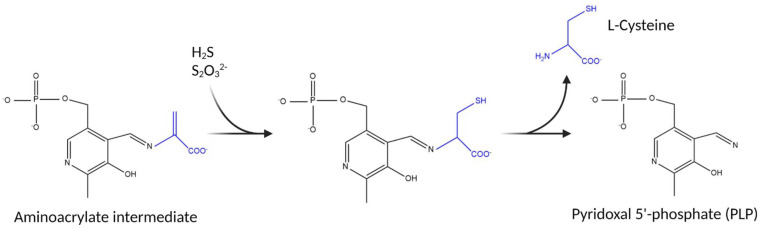
Second half-reaction of PLP-dependent cysteine synthase. Nucleophilic attack by the sulfur donor, shown here as either hydrogen sulfide or thiosulfate, upon the α-aminoacrylate group at the β-carbon position leads to reprotonation of the α-carbon and formation of a cysteine adduct as an external Schiff base. The internal aldimine is subsequently regenerated, releasing free cysteine and completing the overall reaction.

Cysteine synthesis begins with the formation of either O-acetyl-L-serine (OAS) or O-phospho-L-serine (OPS) by serine acetyltransferase (CysE, Cj0763) or phosphoserine aminotransferase (SerC, Cj0326), respectively [9,10]. The resultant metabolites act as sulfur acceptors in the generation of cysteine by either OAS sulfhydrylase (OASS) or OPS sulfhydrylase (OPSS), broadly referred to as cysteine synthase. In bacteria, pyridoxal phosphate (PLP)-dependent cysteine synthase is found in 2 isozymes, CysM and CysK. CysM and CysK share 25–45% identity in peptide sequence but are differentiated by their substrate specificity with respect to the sulfur donor. CysK accepts hydrogen sulfide exclusively as the sulfur donor whereas CysM also accepts thiosulfate [11,12,13]. Cysteine synthase follows a ping-pong enzymatic reaction mechanism, typical of PLP-dependent enzymes (Figure 1) [14,15]. In the resting state, the PLP cofactor forms an internal Schiff base with a catalytic lysine residue. An external Schiff base is then formed between PLP and the sulfur acceptor (OAS or OPS), a β-elimination event occurs releasing either acetate or phosphate and a proton is extracted from the α-position, potentially by the lysine side chain [16]. This forms the α-aminoacrylate intermediate that is covalently linked to PLP. In the second half-reaction (Figure 2), nucleophilic attack by the sulfur donor on the α-aminoacrylate at the β-carbon starts the second half reaction and the α-carbon is subsequently reprotonated [14]; this results in the formation of cysteine bound as an external Schiff base. The internal aldimine is then regenerated releasing cysteine, completing the overall reaction. 

The sole predicted cysteine synthase encoded by *C. jejuni* (Cj0912) is annotated as a CysM-type enzyme but has not been characterised experimentally. In this study, we have determined the substrate specificity of this key enzyme and have further studied the important physiological role of sulfide in driving cysteine synthesis in *C. jejuni*. We also provide evidence that sulfide toxicity for growth is not solely due to inhibition of the cytochrome *c* oxidase. 

## 2. Materials and Methods

### 2.1. Bacterial Strains and Growth Conditions

*C. jejuni* (NCTC 11168) cultures were grown in a MACS-VA500 microaerophilic workstation (Don Whitley Scientific Ltd., Bingley, UK) at 42 °C under microaerobic conditions (10% *v/v* O_2_, 10% *v/v* CO_2_, 80% *v/v* N_2_). *C. jejuni* cells were routinely grown on Columbia agar plates supplemented with 5% *v/v* defibrinated horse blood, or in liquid cultures of Mueller-Hinton broth, supplemented with 20 mM L-Serine. All *C. jejuni* growth media contained 10 µg mL^−1^ vancomycin, with the selective antibiotics kanamycin and apramycin included, where appropriate, at 50 and 60 µg mL^−1^, respectively. Oxygen-limited growth of *C. jejuni* was performed under the following atmosphere: 3% *v/v* O_2_, 10% *v/v* CO_2_, 87% *v/v* N_2_. *E. coli* cultures and plates were routinely grown aerobically at 37 °C in LB medium, with the selective antibiotics kanamycin and ampicillin included, where appropriate, at 50 and 100 µg mL^−1^, respectively.

### 2.2. Heterologous Production and Purification of C. jejuni CysM

*C. jejuni cysM* (*cj0912c*) was amplified from NCTC 11168 genomic DNA with the primers listed in Table S1. The gene fragment included terminal NdeI/XhoI restriction sites, allowing ligation into the T7-inducible *E. coli* expression vector pET28a, such that a hexa-histidine N-terminal tag was introduced for downstream purification by nickel-affinity chromatography. The sequence-verified expression vector was introduced into *E. coli* BL21 (DE3) by selection on kanamycin. LB cultures, supplemented with 100 µM pyridoxal 5′-phosphate (PLP), were grown to an OD600 nm of 0.6 at 37 °C, then transferred to 18 °C and induced with 0.4 mM (final concentration) IPTG. After 20 h, cultures were harvested and cells disrupted by two passes through a chilled French Press under a pressure of 18,000 psi. Lysates were clarified and filtered (0.45 µm pore size), then recombinant CysM was purified using a 5 mL HisTrap HP column (GE Healthcare, Chicago, IL, USA) on an Akta Prime Plus system (Amersham Biosciences, Amersham, UK). Fractions containing CysM were visually evident from the strong yellow colour of the PLP cofactor. Pooled fractions were concentrated and buffer exchanged (100 mM Tris-HCl pH 7.4, 50 mM NaCl) using a 10 kDa cut off Vivaspin column (Cytiva, Marlborough, MA, USA). SDS-PAGE was conducted to verify the purity of CysM prior to assays. CysM has an exceptionally low extinction coefficient (ε 1520 M^−1^ cm^−1^) due to an absence of tryptophan residues and only a single tyrosine residue, in spite of its relatively large size (34.5 kDa, 318 AA). Due to difficulties in accurately quantifying protein concentration by traditional methods, we elected to construct a standard curve of PLP absorbance at 412 nm (0–100 µM PLP, R2 = 0.999), which was used to determine protein concentration by the assumption that active protein units are in a 1:1 molar ratio with the bound PLP cofactor. 

### 2.3. Spectral Analysis of CysM First Half Reaction

UV-Vis absorbance spectra and fluorescence emission scans of CysM were recorded in 1 mL volumes within quartz cuvettes using a UV-2501 (Shimadzu, Kyoto, Japan) and Cary-Eclipse spectrophotometer (Varian, Palo Alto, CA, USA), respectively. The assay mix contained 82.4 µM purified CysM, with OPS or OAS added to a final concentration of 1 mM, where indicated. The assay volume was made up to 1 mL with buffer (100 mM Tris-HCl pH 7.4, 50 mM NaCl).

### 2.4. Kinetic Measurements of Cysteine Synthase Activity

Acidified ninhydrin assays [9] were used to measure the rate of cysteine formation by CysM. L-cysteine standards were prepared (0.1, 0.25, 0.5, 0.75 and 1 mM) and a standard curve constructed with each assay (Supplementary Figure S1C). A 1 mL assay mix contained 100 µL 500 mM HEPES, 200 µL 10 mM OPS or OAS, 200 µL of sulfur donor (sodium sulfide or sodium thiosulfate: 50, 40, 30, 20, 15, 10, 5, 4, 3, 2, 1 mM final) and 500 µL 2 µM CysM protein (diluted in 500 mM HEPES: 1 µM final concentration). The assay mix was incubated in a thermomixer at 37 °C for 60 s, after which 75 µL aliquots were extracted into 15 µL 20% *v/v* TCA to arrest the reaction. 10 µL 100 mM DTT, prepared in 17 mM NaOH, was added and incubated at room temperature for 30 min to reduce any oxidised forms of cysteine. 10 mL acidified ninhydrin reagent was freshly prepared by dissolving 250 mg ninhydrin in 6 mL glacial acetic acid and 4 mL 30% hydrochloric acid. 100 µL acetic acid and 100 µL of the freshly prepared acidified ninhydrin reagent were added to the assay mix and transferred to ice for 2 min. The tubes were centrifuged at 16,000× *g* for 2 min and 50 µL aliquoted in quadruplet into an optical 96-well plate. Finally, 150 µL 95% ethanol was added and mixed well. The amount of L-cysteine formed was determined spectrophotometrically at OD560 nm in a SpectraMax M2 plate reader (Molecular Devices, San Jose, CA, USA). Cysteine synthase specific activity was determined in units of µmol min^−1^ mg^−1^. 

### 2.5. Construction of C. jejuni Mutant Strains

Knock-out deletion mutants of *cysM* (*cj0912c*), *cysE* (*cj0763c*) and *serA* (*cj0891c*) were generated by replacing the majority of the open reading frame with an antibiotic resistance cassette. Mutation plasmids were generated by an isothermal assembly method, as follows. Upstream and downstream target gene flanks were amplified from *C. jejuni* NCTC 11168 genomic (g)DNA, and a kanamycin resistance gene was amplified from the plasmid pJMK30, using the primers listed in Table S1. Backbone plasmid pGEM3ZF was linearised by HincII digestion. These 4 fragments were combined with the HiFi DNA assembly master mix (New England Biolabs, Ipswich, MA, USA) and incubated at 50 °C for 1 h. The cloning design is such that recombination occurs specifically between homologous ends of complementary fragments, resulting in a plasmid that contains the kanamycin resistance cassette inserted between the 2 gene flanks. The resultant DNA was transformed into *E. coli* DH5α and transformants were selected for dual ampicillin/kanamycin resistance. Plasmid DNA isolated from clones and confirmed to be correct by automated Sanger sequencing (Eurofins) was used to transform wildtype *C. jejuni* NCTC 11168 by electroporation. Transformants were plated onto selective media and kanamycin resistant clones were isolated. Successfully generated mutants were identified by PCR screening to confirm correct recombination into the genome. 

### 2.6. Construction of C. jejuni Complement Strains

In the case of the Δ*cysM* and Δ*cysE* mutants, genetic complementation was performed to verify the mutant phenotype. The pRRA plasmid system was used to introduce a copy of the deleted gene back into the genome at a distal rRNA locus, under the control of a constitutive promoter [17]. Briefly, genes were amplified from *C. jejuni* NCTC 11168 gDNA using the primers listed in Table S1, which include adaptors homologous to the terminal ends of XbaI/MfeI digested pRRA. These 2 fragments were combined by isothermal assembly, as described above. This results in gene insertion into pRRA, in such an orientation as to be driven by the constitutive promoter in the plasmid. *E. coli* transformants were selected for apramycin resistance and correct insertion verified by PCR screening and sequencing. *C. jejuni* mutant strains were transformed with their respective complementation plasmid, as above, selecting for kanamycin/apramycin resistant clones.

### 2.7. C. jejuni Growth Assays on Sulfur Limited Media

A defined media (DM) based on MEM was developed without any usable sulfur sources for *C. jejuni* (Table S2). Growth in this media was dependent on the addition of a defined sulfur source, verified in each instance with a sulfur-negative control. *C. jejuni* starter cultures were grown in MHS broth supplemented with 2 mM L-cysteine, from which inoculums were prepared by multiple washes in PBS. 30 mL volumes of DM in 100 mL conical flasks were inoculated to an OD600 nm of 0.1 and initial growth rates followed over the course of 6 h with 140 rpm shaking under microaerobic conditions. Sulfur sources were either added in total at the start of the growth curve, or added incrementally each hour, as indicated. Growth assays were performed in biological triplicate, and the OD600 nm was recorded every hour.

### 2.8. Oxidase Activity Measurements by Oxygen Electrode

Cells were grown as indicated for the relevant activity measurements. Cells harvested from media were washed several times in PBS and finally resuspended in a 1 mL volume of PBS. A Clark-type oxygen electrode equipped with a 2 mL chamber (Rank Brothers Ltd., Cambridge, UK) connected to a Lab-Trax-4/16 with LabScribe software (World Precision Instruments, Sarasota, FL, USA) was used to record oxygen consumption rates, calibrated to 220 nmol dissolved O_2_ mL^−1^ for air saturated buffer at 42 °C. Total oxidase activity was recorded, using 20 mM final sodium formate as electron donor. Total protein concentration of the cell suspensions was determined by Lowry assay and specific oxidase activity determined as nmol oxygen consumed min^−1^ mg^−1^ total protein.

## 3. Results

### 3.1. Substrate Specificity of the C. jejuni Cysteine Synthase, Cj0912

**Figure 3:**
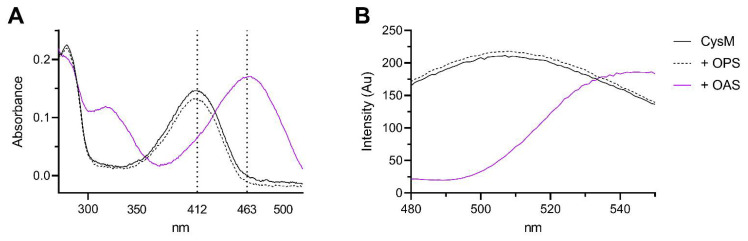
First half-reaction of purified CysM with sulfur acceptors OPS and OAS. (**A**) UV-vis scan of CysM generating the aminoacrylate intermediate (463 nm maximum) and reduction in PLP absorbance (412 nm maximum) with OAS, but not OPS. (**B**) A large shift in the emission spectra of the CysM PLP cofactor (excited at 412 nm) occurs with the addition of OAS, but not OPS.

In order to investigate the substrate specificity of *C. jejuni* cysteine synthase, Cj0912 was heterologously produced and purified from *E. coli*, utilising a hexa-histidine tag and nickel affinity chromatography, as described in detail in Section 2.2. An SDS-PAGE gel showing purification of Cj0912 can be viewed in Supplementary Figure S1A. Cj0912 purified with the PLP cofactor in situ, clearly evident by the yellow colour of the purified protein. UV-Vis scans revealed the characteristic absorbance spectrum of PLP, with a broad peak centred at 412 nm maximum (Figure 3A). We utilised the PLP absorbance characteristics in Figure 1 to differentiate resting PLP and the aminoacrylate intermediate formed upon reaction with a sulfur acceptor. Absorbance spectra were recorded before and after addition of the candidate sulfur acceptors OAS or OPS to purified Cj0912 (Figure 3A). A shift in the absorbance maximum from 412 nm to 463 nm was clearly evident with OAS, but no change occurred with OPS. This experiment was repeated with the fluorescence emission spectrum of the PLP cofactor monitored when the enzyme was excited at 412 nm (Figure 3B). Consistent with the absorbance measurements, a significant shift in the emission spectrum was evident with OAS, but not OPS. These results clearly demonstrate that *C. jejuni* cysteine synthase solely utilises OAS as sulfur donor. 

**Figure 4:**
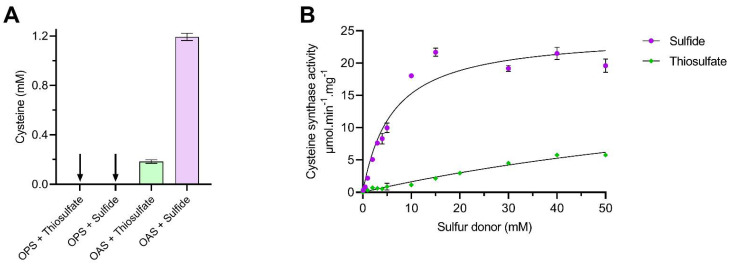
Cysteine synthase activity of CysM with sulfur donors thiosulfate and sulfide. (**A**) Synthesis of cysteine by CysM was followed by acid-ninhydrin assay. After 30 min, cysteine was detected for both thiosulfate and sulfide when using OAS as acceptor, but not OPS. (**B**) Michaelis-Menten kinetics for sulfide and thiosulfate when using OAS as sulfur acceptor. Sulfide: *K*_m_ 6.12 ± 1.12, *V*_max_ 24.60 ± 1.46. Thiosulfate: *K*_m_ 118.1 ± 68.7, *V*_max_ 20.80 ± 9.26.

The second half reaction involves attack by a sulfur donor upon the aminoacrylate intermediate to generate and release free L-Cysteine, as described in Figure 2. We analysed the utilisation of sulfide and thiosulfate as sulfur donors by an acidified ninhydrin method to measure the rate of L-Cysteine formation by purified Cj0912 (Figure 4). We first measured the production of L-Cysteine when Cj0912 was provided with an excess combination of OAS or OPS and sulfide or thiosulfate (Figure 4A). No L-Cysteine was produced when OPS was provided as sulfur acceptor. With OAS as sulfur acceptor, L-Cysteine was produced with both sulfur donors, though markedly less with thiosulfate than sulfide. To determine the relative rates with the two sulfur donors, we analysed the Michaelis-Menton kinetics of the enzyme over a range of sulfur donor concentrations, in the presence of excess OAS (Figure 4B). Sulfide proved to be a far superior sulfur donor, while thiosulfate was such a poor donor that reliable *K*_m_ and *V*_max_ values could not be calculated. Taken together, we conclude sulfide to be the physiological sulfur donor for Cj0912, while retaining some limited ability to utilise thiosulfate. The substrate specificity of Cj0912 as defined here confirms its annotation as a CysM-type cysteine synthase. 

### 3.2. Sulfide as an Effective Sole Sulfur Source for C. jejuni

**Figure 5:**
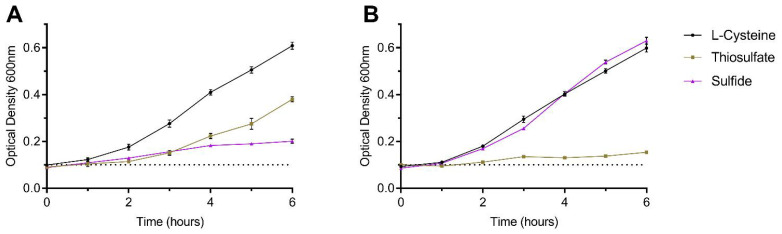
Growth of *C. jejuni* NCTC11168 wildtype on defined sulfur sources. Sulfur deficient defined media with individual sulfur sources added to either (**A**) 2 mM final, added in total at T0, or (**B**) 0.1 mM total, added progressively every hour over the course of 6 h. The inoculum level is indicated by the dashed line.

A series of experiments were conducted in a sulfur-limited defined medium to analyse the ability of *C. jejuni* to grow on individual sulfur sources. Vorwerk et al. [8] previously defined sulfur source utilisation by *C. jejuni* in detail, including growth on sulfide and thiosulfate. Our results are in agreement: when provided at a relative excess (2 mM), growth on either thiosulfate or sulfide is poor compared to L-Cysteine (Figure 5A). However, we predicted that poor growth on sulfide may occur as a high concentration of substrate is typically added to the inoculum in bolus at the beginning of the growth curve. This differs from the more likely situation in a host where sulfide is continuously produced at a lower level. To mimic this, we conducted growth experiments wherein small amounts of sulfur source were progressively added to the culture each hour (Figure 5B). In the case of sulfide, 0.1 mM total addition over the course of 6 h was determined to support maximum growth, comparable to that of L-Cysteine. Notably, thiosulfate could barely stimulate growth under these conditions. We therefore conclude that sulfide can be an effective sulfur source for *C. jejuni* when it is at a level that is not toxic to the cell. 

**Figure 6:**
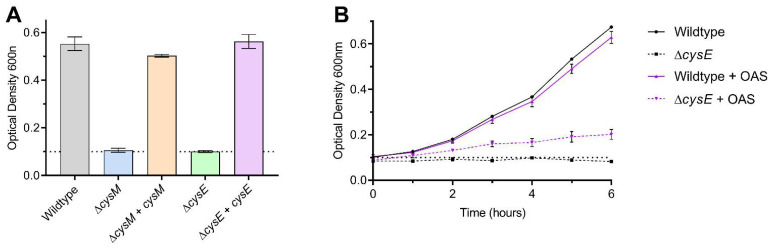
Growth of *cysM* and *cysE* mutants on sulfide. (**A**) Growth of wildtype, Δ*cysM*, Δ*cysE* and genetically complemented strains in defined media with sulfide as the sole sulfur source, displayed as the optical density reached after 6 h. (**B**) Chemical complementation of the OAS deficient *cysE* mutant strain in defined media with sulfide as the sole sulfur source, by the exogenous addition of OAS. The inoculum level is indicated by the dashed lines.

To define the pathway requirements for growth on sulfide as sole sulfur source, we constructed mutant strains of *C. jejuni* NCTC 11168 in *cysM* (cysteine synthase; *cj0912c*) and *cysE* (serine acetyltransferase; deficient in OAS synthesis; *cj0763c*). Neither mutant was able to utilise sulfide (Figure 6A) or thiosulfate (Supplementary Figure S2D) for growth, but both grew normally on L-Cysteine (Figure 6A, Supplementary Figure S2A–C). These phenotypes were restored to wildtype levels by genetic complementation (Figure 6A) and, in the case of Δ*cysE*, partially by chemical complementation through exogenous addition of OAS (Figure 6B). 

### 3.3. Sulfide Toxicity in C. jejuni Is Not Solely Attributed to Inactivation of the Terminal Cytochrome C Oxidase

**Figure 7:**
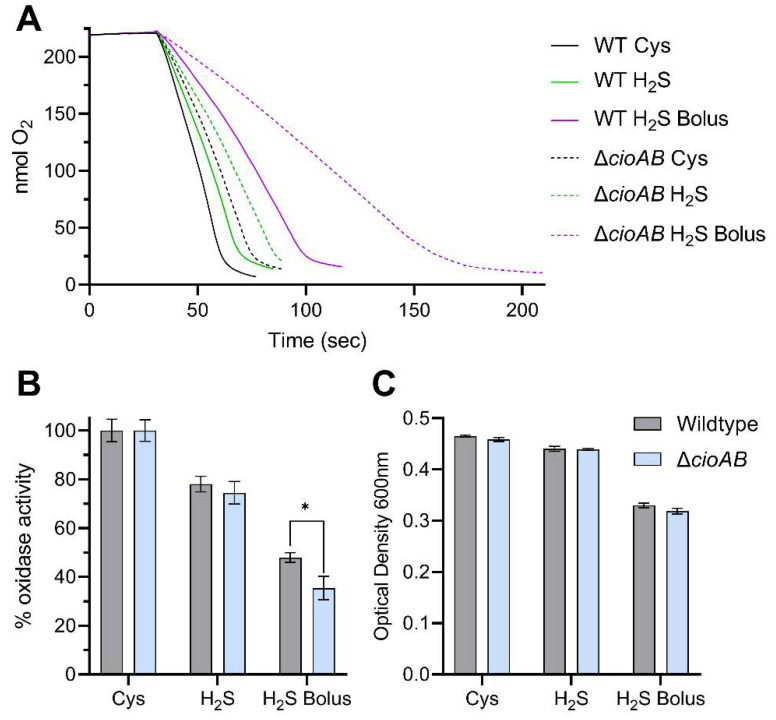
Inactivation of terminal oxidase activity by sulfide. (**A**) Total oxidase activity of wildtype and *cioAB* mutant with sodium formate as electron donor, measured in a Clark-type oxygen electrode. Cells were grown with either L-Cysteine or sulfide as sole sulfur source. Sulfide was added either progressively throughout the growth curve or by bolus addition at the start (0.1 mM final). (**B**) Total oxidase activity of the above samples, displayed as percentage activity relative to the L-Cysteine control. * *p* < 0.05. (**C**) Growth of the above samples, displayed as the optical density reached after 4 h of growth.

We showed above that growth on sulfide is poor when the concentration added to cultures is too high and becomes toxic to the cell. We have previously shown that the primary terminal oxidase in *C. jejuni*, the *cbb*_3_-type cytochrome *c* oxidase CcoNOPQ, is sensitive to inactivation by sulfide [18]. *C. jejuni* encodes a second, lower affinity oxidase, CioAB (a type of cytochrome *bd* oxidase), which is largely sulfide insensitive. Here, we measured microaerobic growth and oxidase activity of both wildtype *C. jejuni* and an isogenic Δ*cioAB* mutant (in which the sulfide sensitive CcoNOPQ is the only oxidase) in defined media with sulfide added either by bolus or progressively, as described above (Figure 7). Total oxidase activity was measured in whole cells using an oxygen electrode with formate as electron donor (Figure 7A). Progressive addition of sulfide resulted in a 20–25% reduction in total oxidase activity, relative to the cysteine control, in both wildtype and Δ*cioAB* (Figure 7B), with little impairment of growth (Figure 7C). When 0.1 mM sulfide was added in bolus, total oxidase activity was greatly impaired, to 48% of the control rate in wildtype, and 35% in Δ*cioAB* (Figure 7B). Notably, despite the greater sulfide sensitivity of Δ*cioAB* (in terms of oxidase activity), and the specific oxidase activity of bolus sulfide treated Δ*cioAB* being just 55% that of the corresponding wildtype cells (Supplementary Figure S3B), growth inhibition by sulfide was not significantly different between the two strains (Figure 7C). 

**Figure 8:**
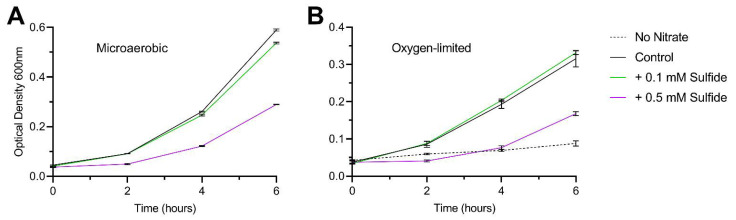
Growth inhibition of *C. jejuni* in complex media by sulfide. Cells were grown in Muller-Hinton broth supplemented with 20 mM L-Serine and 10 mM sodium nitrate, under either (**A**) microaerobic or (**B**) oxygen-limited conditions. Sulfide was added by bolus addition at the beginning of the growth curve at either 0.1 or 0.5 mM final. No sulfide controls were included in both conditions, and a nitrate minus control included under oxygen-limited conditions to confirm nitrate dependent growth and oxygen limitation.

In this experiment, cells were grown microaerobically in defined media with no terminal electron acceptors available besides oxygen. It is surprising then that, in the presence of sulfide, while oxidase activity does correlate with growth inhibition, it is clearly not the limiting factor for achieving maximum growth rate. To further test whether sulfide toxicity is primarily due to factors independent of oxidase inactivation, we conducted a set of growth curves with the wildtype strain under both microaerobic and oxygen-limited conditions, with sulfide added at either 0.1 or 0.5 mM (Figure 8). The cells were grown in rich broth supplemented with 10 mM sodium nitrate in order to support oxygen-limited growth with an alternative electron acceptor. Under a microaerobic atmosphere, growth was slightly inhibited by 0.1 mM sulfide and greatly inhibited by 0.5 mM sulfide (Figure 8A). Under oxygen-limited conditions, the growth rate is much reduced compared to microaerobic conditions, but the relative inhibition pattern by sulfide is similar, albeit no inhibition was observed at the lower sulfide concentration (Figure 8B). Taken together, these data demonstrate that inhibition of terminal oxidase activity has only a minor contribution towards the inhibition of growth by sulfide in *C. jejuni*.

## 4. Discussion

In this work we experimentally confirm that the *C. jejuni* PLP-dependent cysteine synthase Cj0912 is of the CysM type, specifically utilising OAS as sulfur acceptor and able to use both sulfide and thiosulfate as the sulfur donor. However, we found thiosulfate to be a poor sulfur donor for purified Cj0912 and this was supported by very poor growth of *C. jejuni* on thiosulfate as a sole source of sulfur (Figure 4B and Figure 5). Thiosulfate is generated by the host epithelium to reduce toxic sulfide levels and so it is perhaps surprising that *C. jejuni* does not readily utilise this substrate for cysteine synthesis [19]. In addition to poor enzymatic rates of CysM with thiosulfate, the *C. jejuni* genome does not encode the typical ABC-type thiosulfate transporter [20]. Whether a specific thiosulfate transporter exists in *C. jejuni* is unknown; its apparent absence may explain why such high exogenous concentrations are required to support even modest growth. Some *C. jejuni* strains possess a bi-functional tetrathionate reductase, TsdA, which can oxidise thiosulfate to tetrathionate for energy conserving oxygen-linked respiration [21]. It is possible that thiosulfate may be preferentially utilised in vivo for this purpose, a process not requiring active transport as TsdA is periplasmic. 

**Figure 9:**
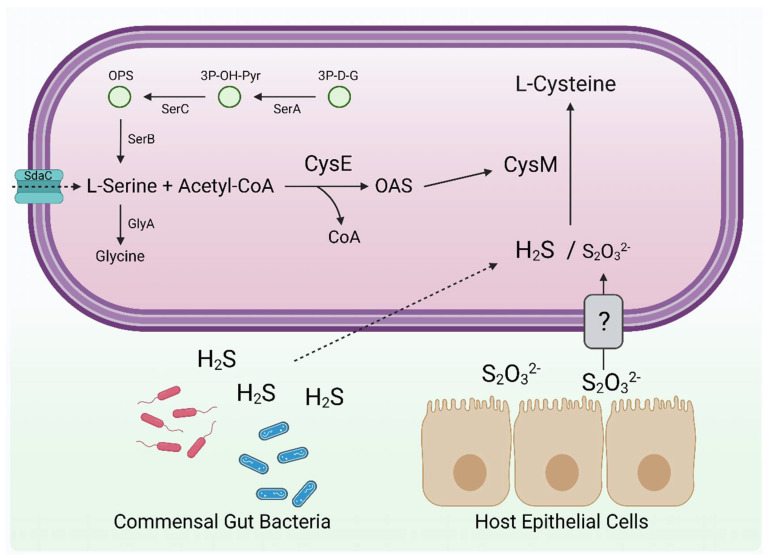
Model for cysteine synthesis in *C. jejuni***:** *C. jejuni* cysteine synthase (CysM) exclusively utilises OAS as sulfur acceptor, which is generated by CysE in an acetyl-CoA dependent reaction from L-Serine. L-Serine can be directly imported from the environment by the transporter SdaC, or endogenously synthesised from 3-Phospho-D-Glycerate (3P-D-G) by SerABC. CysM can utilise both sulfide and thiosulfate as sulfur donors, although the *C. jejuni* enzyme has a clear enzymatic preference for sulfide. Both are abundant in the gut; sulfide is generated from host microbiota and thiosulfate by host epithelial cells. Hydrogen sulfide can freely diffuse across membranes while thiosulfate requires active transport. ?: thiosulfate transporter, yet to be identified in *C. jejuni*. Figure created with BioRender.com.

We observed that when purified CysM was incubated with OAS alone, formation of the aminoacrylate intermediate was transient, and OAS appeared to be turned over by the enzyme in a concentration dependent manner (Supplementary Figure S1B). The presumed release of a free acrylate moiety by CysM in the absence of a usable sulfur donor could contribute towards reactive aminoacrylate toxicity, for which *C. jejuni* encodes 2 putative deaminases [22]. By contrast, OPS did not function as a sulfur acceptor for CysM. OPS is synthesised from 3-Phospho-D-Glycerate by the SerABC pathway and used to generate L-Serine (Figure 9). We initially attempted to generate an OPS-deficient strain by mutating *serC*, encoding 3-phosphoserine aminotransferase. We observedthis mutant to have a severe generic growth defect, irrespective of the media supplementation used. In this mutant, the intermediate 3-phosphohydroxypruvate (3P-OH-Pyr) is presumed to accumulate as no other pathways are available to metabolise it. There is no known toxicity route for 3P-OH-Pyr in bacteria, yet the subsequently generated Δ*serA* strain had only a minor growth defect, despite also being OPS deficient and a serine/glycine auxotroph (Figure 9; Supplementary Figure S3A). This interesting observation suggests 3P-OH-Pyr accumulation is highly toxic to *C. jejuni* through an as yet unknown mechanism. We also observed that the serine auxotrophy of Δ*serA* could not be complemented by supplementation with excess glycine, demonstrating that the *C. jejuni* serine hydroxymethyltransferase GlyA is not bidirectional, as described for some bacteria (Figure 9; Supplementary Figure S3A) [23]. Supplementation with excess L-Serine largely recovered the auxotrophy of Δ*serA* but there was a small reduction in growth compared to wildtype (Supplementary Figure S3A). This observation has been made previously in *C. jejuni*, with the demonstration that imported L-Serine is preferentially degraded as an energy source rather than utilised for protein synthesis [24]. 

Sulfide is generated in the gut by the microbiota, primarily the abundant sulfate-reducing bacteria [25,26]. In this work, sulfide is clearly shown to be the preferential sulfur source for the *C. jejuni* CysM and to act as an excellent sole sulfur source to support growth (Figure 4B and Figure 5B). Sulfide has historically been regarded as a toxic substance with strong anti-microbial activity [27,28]. Measurements of sulfide concentration in the gut range between 0.2–2 mM [29]. Even the lower end of these estimates would prove severely inhibitory to *C. jejuni*, yet it is a highly successful enteric pathogen. More recently it has been demonstrated that the majority of sulfide is in a bound form with fecal components, and that free sulfide gas comprises just 8% of total sulfide [29,30]. Based on the previous range, this would bring sulfide levels that enteric bacteria encounter in the free form down to the low micromolar range, wherein it would be sub-inhibitory and function as an ideal sulfur source for *C. jejuni*. This may reflect the historic portrayal of sulfide as a poor sulfur source in laboratory experiments due to the methodology used to assay growth on sulfide, as we have demonstrated (Figure 5). 

At the onset of this study, we assumed the mechanism of sulfide toxicity in *C. jejuni* would be largely through inactivation of the primary terminal oxidase, CcoNOPQ. We have demonstrated previously that the secondary terminal oxidase, CioAB, is largely sulfide insensitive [18]. Surprisingly, there was little difference between wildtype *C. jejuni* and the Δ*cioAB* mutant in terms of growth on sulfide or reduction in relative total oxidase activity (Figure 7). At the physiologically relevant concentrations of sulfide tested, this brings into question the role of CioAB in sulfide resistance and further work is required to assign the physiological function of CioAB, which in other enteric bacteria has been suggested to allow continued respiration in the presence of sulfide [31,32]. In addition, despite a >20% reduction in oxidase activity in cells grown on sulfide, the growth rate was barely impacted compared to growth on L-Cysteine, suggesting oxidative respiration is not the rate limiting factor in the microaerobic growth of *C. jejuni* (Figure 5B and Figure 7). As such, although growth rate was correlated with total oxidase activity in sulfide treated cells, we questioned whether this was the causal mechanism. To address this, we performed oxygen-limited growth assays where growth was dependent on nitrate reduction as an alternative electron acceptor to oxygen (Figure 8). Under these conditions, where respiration with oxygen is irrelevant to growth rate, we found growth inhibition by sulfide was relatively equal to that of microaerobic cultures. This suggests that sulfide targets other than CcoNOPQ are responsible for the majority of the observed growth inhibition. 

Sulfide is known to specifically target heme-proteins, reduce metal centres and generate sulfo-adducts [33]. Since these modifications are often deleterious, the high abundance of cytochromes in *C. jejuni* provide an obvious mechanism for the antimicrobial activity of sulfide at high concentrations. Given *C. jejuni* efficiently utilises sulfide at physiologically relevant concentrations with minimal growth inhibition, and that sulfide is a prominent indicator of reaching the colonisation niche, it seems plausible that enteric pathogens like *C. jejuni* that do not produce sulfide endogenously could detect exogenous sulfide to switch to a pathogenic lifestyle. Indeed, eukaryotes utilise sulfide as a controlled signalling molecule to induce various processes including neuro-modulation and muscle relaxation [34]. However, whether any signalling processes occur in *C. jejuni* in response to sulfide is currently unknown, and a warranted avenue of future investigation.

## Data Availability

Not applicable.

## Supplementary Materials

The following supporting information can be downloaded at: https://www.mdpi.com/article/10.3390/biom13010086/s1, Figures S1–S3: SDS-PAGE of purified CysM; aminoacrylate intermediate turnover; L-Cysteine standard curve for ninhydrin assay; full sulfur-limited growth curve data; *serA* serine/glycine auxotrophic growth; specific total oxidase activity (absolute rates).; Table S1: Primers used in this study; Table S2: Sulfur limited defined media used in this study.
