# Supplementary material for: Cysteine Biosynthesis in Campylobacter jejuni: Substrate Specificity of CysM and the Dualism of Sulfide
Source: Biomolecules. 2022 Dec 31;13(1):86. doi: 10.3390/biom13010086 (PMC9855970; doi:10.3390/biom13010086)
Supplement: Supplementary file 1 [file biomolecules-13-00086-s001.zip › biomolecules-2131951-supplementary.pdf]

biomolecules

MDPI

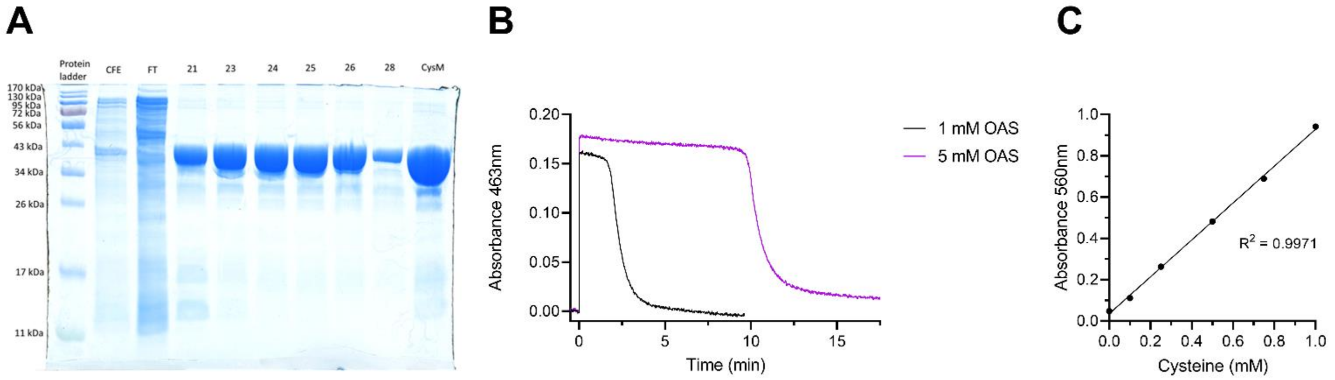

Figure S1. (**A**) SDS-PAGE showing nickel affinity purified CysM protein. CFE: cell free extract; FT: Ni-column flow through; 21-28: Ni-column elution fractions; CysM: Buffer exchanged purified CysM protein. (**B**) Degradation of the aminoacrylate intermediate of purified CysM in the absence of sulfur acceptor is dependent on the concentration of sulfur acceptor and indicates spontaneous turnover. (**C**) Standard curve of cysteine used in the acid ninhydrin assays.

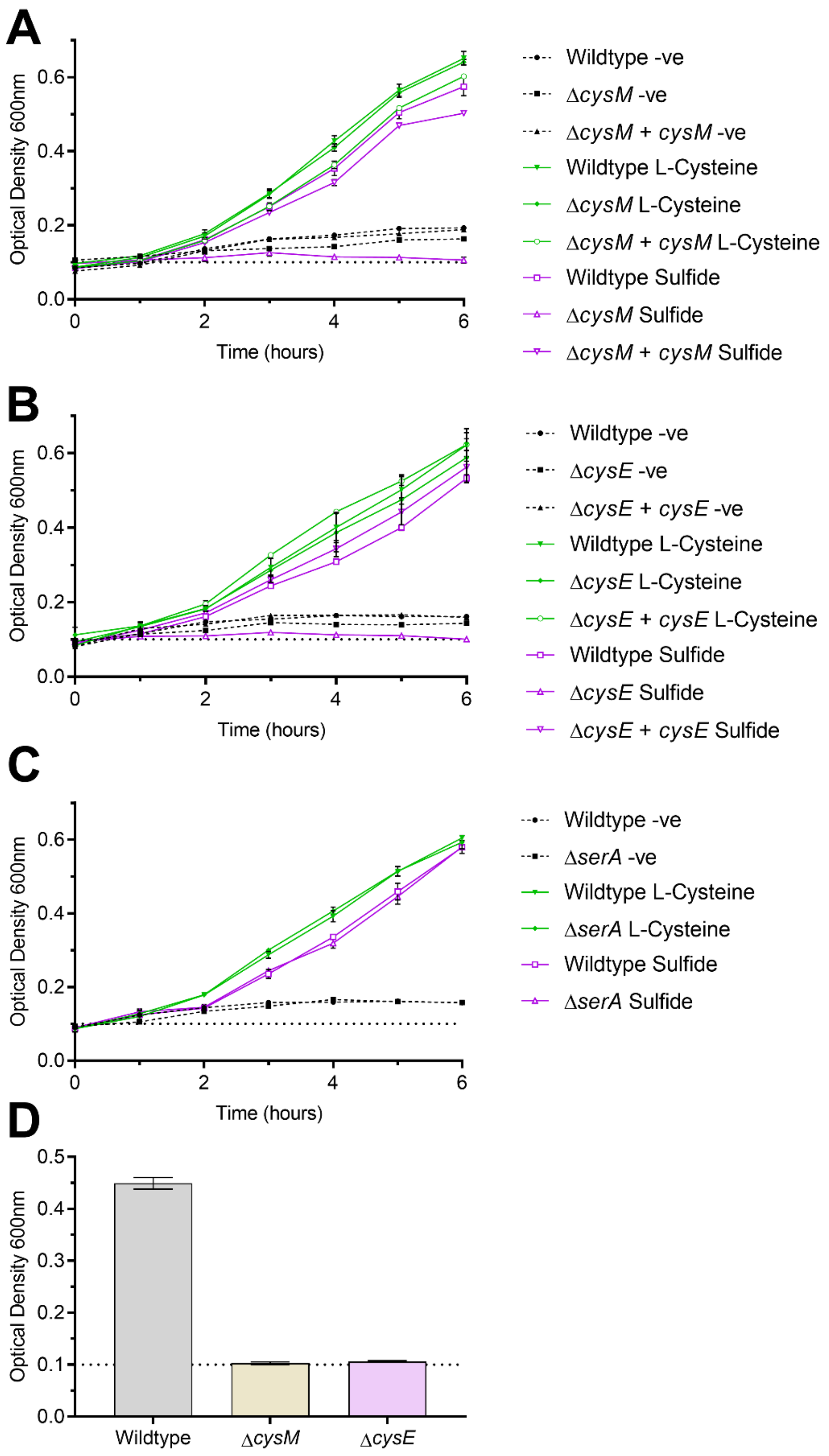

Figure S2. Full growth curves in sulfur deficient defined media with either no addition (-), cysteine or sulfide added, for wildtype and (**A**) Δ*cysM* and genetically complemented strain, (**B**) Δ*cysE* and genetically complemented strain and (**C**) Δ*serA*. (**D**) Growth of wildtype, Δ*cysM* and Δ*cysE* with thiosulfate as sole sulfur source, displayed as the optical density reached after 6 h. In all panels, the inoculum level is indicated by the dashed line.

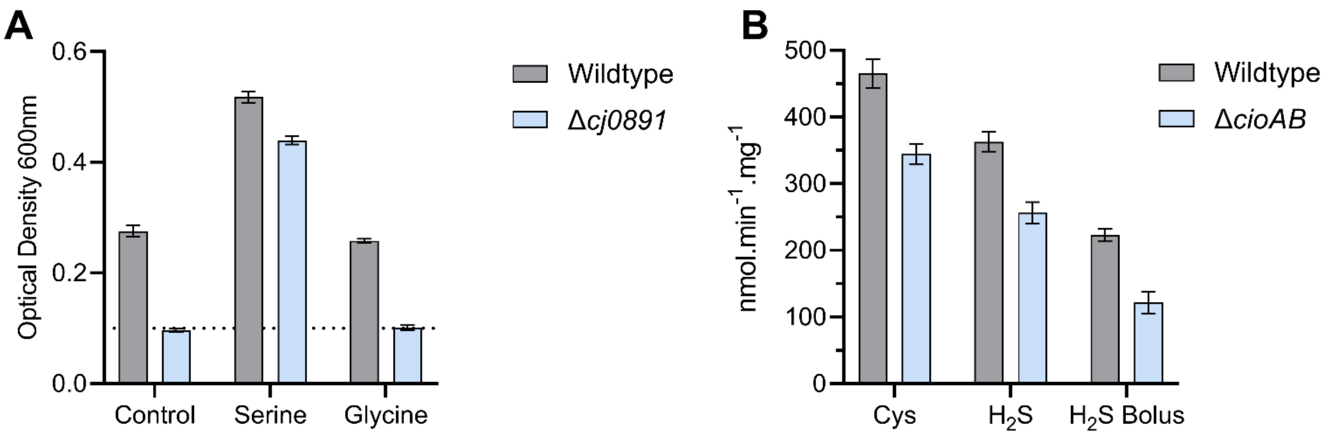

Figure S3. (**A**) Growth of *C. jejuni* NCTC11168 wildtype and *serA* (*cj0891*) mutant on L-Serine minus defined media with the addition of either L-Serine or glycine (10 mM final), displayed as the optical density at 600nm achieved after 6 h. Dotted line indicates the inoculum level. (**B**) Total oxidase specific activity, calculated in nmol oxygen consumed, per minute, per mg of total protein (see Figure 7).

Table S1. Primers used in this study.

**Primer name**	**Sequence 5′–3′**	**Notes**
pET280912 fwd	CAGTCATATGAAAGTACATGAAAAAGTAAGTGAG	Amplification of *cj0912c* for insertion into pET28a
pET280912 rev	ATGCCTCGAGTTAAGCAAAAAGATCGGTTGAG	
KanR fwd	ATTCTCCTTGGTTCTCATGTTTGACAGCTTAT	Amplification of KanR from pJMK30
KanR rev	GCACACCTTGGCTAGGTACTAAAACAATTCAT	“
0912F1	GAGCTCGGTACCCGGGGATCCTCTAGAGTCATTAGTTGCTCAAACAGCTG	Amplification of downstream flank of *cj0912c*
0912R1	AAGCTGTCAAACATGAGAACCAAGGAGAATCACAAATCATAGCCAAAGAA	“
0912F2	GAATTGTTTTAGTACCTAGCCAAGGTGTGCAGGTATATCAAGTGGAGCAA	Amplification of upstream flank of *cj00912c*
0912R2	AGAATACTCAAGCTTGCATGCCTGCAGGTCGTCCGGCAACTCTTAGTTTA	“
0763F1	GAGCTCGGTACCCGGGGATCCTCTAGAGTCGCGATGGAGAAATT	Amplification of downstream flank of *cj0763c*
0763R1	AAGCTGTCAAACATGAGAACCAAGGAGAATATTCCGCTAATCATTCTAGC	“
0763F2	GAATTGTTTTAGTACCTAGCCAAGGTGTGCCATTAGAGCCATAGATGCAA	Amplification of upstream flank of *cj00763c*

0763R2	AGAATACTCAAGCTTGCATGCCTGCAGG TCGTGTCAAAATCAGGTTCACC	"
0891F1	GAGCTCGGTACCCGGGGATCCTCTAGAG TCTAAGCACTTCTATGCTTGAAA	Amplification of downstream flank of *cj0891c*
0891R1	AAGCTGTCAAACATGAGAACCAAGGAG AATGCAACTTCAATATCGCTTAG	"
0891F2	GAATTGTTTTAGTACCTAGCCAAGGTGTG CCAGTGTTTCAGGAACAGTGT	Amplification of upstream flank of *cj0891c*
0891R2	AGAATACTCAAGCTTGCATGCCTGCAGG TCGCTCATTGAAATCAAAAGGT	"
pRRA0912 fwd	AATAATGACCCGGGGATCCTCTAGAAAA AAGGATATTAAAATTATGAAAGTACAT	Amplification of *cj0912c* for complementation plasmid pRRA
pRRA0912 rev	TTGAAATCTAAACAAGAATCAATTGGCT TTATCTTATTGATGAAAAAGG	"
pRRA0763 fwd	AATAATGACCCGGGGATCCTCTAGAGCA TAGGAGTTTAGATGAATTTT	Amplification of *cj0763c* for complementation plasmid pRRA
pRRA0763 rev	TTGAAATCTAAACAAGAATCAATTGGCA GAAAATTGCATTTTTAC	"

Table S2. Sulfur limited defined media used in this study.

**Inorganic salts**	**mM final**
Magnesium Sulfate (MgSO4.7H2O)	1
Potassium Chloride (KCl)	5
Sodium Bicarbonate (NaHCO3)	15
HEPES	15
Sodium Chloride (NaCl)	120
Sodium Phosphate monobasic (NaH2PO4-H2O)	1
Ammonium sulfate	5
**Amino acids**	**mM final**
L-Glycine	2
L-Glutamate K salt	2
L-Aspartate K salt	2
L-Alanine	2

L-Arginine hydrochloride	2
L-Asparagine.H2O	2
L-Tyrosine disodium salt dihydrate	2
L-Histidine hydrochloride-H2O	2
L-Isoleucine	2
L-Leucine	2
L-Lysine hydrochloride	2
L-Phenylalanine	2
L-Threonine	2
L-Valine	2
L-Proline	2
L-Tryptophan	2
L-Methionine	2
L-Serine	5
L-Glutamine	5
**Other**	**μM final**
Sodium Formate	5000
Sodium Pyruvate	5000
Niacinamide	50
D-Calcium pantothenate	10
Vitamin B12	1
i-Inositol	10
Biotin	10
Folic acid	10
Pyridoxal hydrochloride	10
Riboflavin	10

Thiamine hydrochloride	10
Sodium Metabisulphite	10
Ferrous Sulphate.7H2O	25
Ascorbic Acid	50
**Metals**	**μM final**
EDTA disodium .2H2O	10
Boric acid	0.1
CuSO4.5H2O	1
ZnCl2	1
MnCl2.4H2O	1
CoCl2.6H2O	1
NiCl2.6H2O	1
Na2MoO4.2H2O	1
Na2WO4 · 2H2O	0.1
Sodium Selenite	0.1
